# A comprehensive and clinical‐oriented evaluation criteria based on DVH information and gamma passing rates analysis for IMRT plan 3D verification

**DOI:** 10.1002/acm2.12910

**Published:** 2020-05-20

**Authors:** Xin Yi, Wen‐li Lu, Jun Dang, Wei Huang, Hai‐xia Cui, Wan‐chun Wu, Ying Li, Qing‐feng Jiang

**Affiliations:** ^1^ Department of Oncology The First Affiliated Hospital of Chongqing Medical University Chongqing China

**Keywords:** action levels, dosimetric verification, DVH information, gamma passing rates, quality assurance

## Abstract

**Purpose:**

To accomplish the 3D dose verification to IMRT plan by incorporating DVH information and gamma passing rates (GPs) (DVH_GPs) so as to better correlate the patient‐specific quality assurance (QA) results with clinically relevant metrics.

**Materials and methods:**

DVH_GPs analysis was performed to specific structures of 51 intensity‐modulated radiotherapy (IMRT) treatment plans (17 plans each for oropharyngeal neoplasm, esophageal neoplasm, and cervical neoplasm) with Delta4 3D dose verification system. Based on the DVH action levels of 5% and GPs action levels of 90% (3%/2 mm), the evaluation results of DVH_GPs analysis were categorized into four regions as follows: the true positive (TP) (%DE> 5%, GPs < 90%), the false positive (FP) (%DE ≤ 5%, GPs < 90%), the false negative (FN) (%DE> 5%, GPs ≥ 90%), and the true negative (TN) (%DE ≤ 5%, GPs ≥ 90%). Considering the actual situation, the final patient‐specific QA determination was made based on the DVH_GPs evaluation results. In order to exclude the impact of Delta4 phantom on the DVH_GPs evaluation results, 5 cm phantom shift verification was carried out to structures with abnormal results (femoral heads, lung, heart).

**Results:**

In DVH_GPs evaluation, 58 cases with FN, 5 cases with FP, and 2 cases with TP were observed. After the phantom shift verification, the extremely abnormal FN of both lung (%*DE* = 21.52%±8.20%) and heart (%*DE* = 19.76%) in the oropharyngeal neoplasm plans and of the bilateral formal heads (%*DE* = 26.41%±13.45%) in cervical neoplasm plans disappeared dramatically. DVH_GPs analysis was performed to all evaluation results in combination with clinical treatment criteria. Finally, only one TP case from the oropharyngeal neoplasm plans and one FN case from the esophageal neoplasm plans did not meet the treatment requirements, so they needed to be replanned.

**Conclusion:**

The proposed DVH_GPs evaluation method first make up the deficiency of conventional gamma analysis regarding intensity information and space information. Moreover, it improves the correlation between the patient‐specific QA results and clinically relevant metrics. Finally, it can distinguish the TP, TN, FP, and FN in the evaluation results. They are affected by many factors such as the action levels of DVH and GPs, the feature of the specific structure, the QA device, etc. Therefore, medical physicist should make final patient‐specific QA decision not only by taking into account the information of DVH and GPs, but also the practical situation.

## 1 INTRODUCTION

Intensity‐modulated radiation therapy (IMRT) has currently become a radiotherapy technique universally adopted by the radiation therapy centers of countries in the world. The reason is that it is able to generate a therapeutic dose distribution that is highly conformable to the target volume. At the same time, it can provide a better coverage volume for tumor as well as a better protection for the organs at risk. These advantages are achieved by modulating the parameters such as therapeutic dose and the motion speed of the leaves of multileaf collimator (MLC)^1^. Due to the above advantages, IMRT plan requires higher delivery precision of linear accelerator and calculation accuracy of treatment planning system (TPS)^2^. In order to ensure the patient's safety, patient‐specific quality assurance (QA) is an essential key link before delivery of IMRT plan^3^. Patient‐specific QA analyzes and evaluates the deviation between the predicted dose and the measured dose of patient plan in phantom by film dosimetry^4^, ionization chambers^5^, electronic portal imaging device (EPID)^6^, two‐dimensional (2D) array detector^7^, ^8^, ^9^, three‐dimensional (3D) dosimetric systems, and the gel dosimetry^10^, ^11^, etc. For performing quantitative evaluation to the calculation accuracy of TPS system and the delivery precision of linear accelerator, Low et al.^12^ proposed the gamma analysis method. It adopts dimensionless gamma index to incorporate the dose difference (DD) and the distance‐to‐agreement (DTA). That is how it evaluates the difference between the predicted dose distribution and the measured dose distribution within the region of interest (ROI). It was recommended in the AAPM TG‐119 report^13^ that the gamma passing rates (GPs) within the ROI under 3%/3 mm (global normalization and 10% threshold) can be used as the evaluation criteria for the patient‐specific QA in gamma analysis. Subsequently, the gamma criteria were updated to 3%/2mm (global normalization and 10% threshold) in AAPM TG‐218 report^14^. Nowadays, this evaluation criterion has become a widely accepted standard for all major radiotherapy centers, and has also been widely used in QA analysis software of major manufacturers.

From the clinical treatment point of view, the analysis results of patient‐specific QA should be correlated with clinically relevant metrics (such as the estimated deviations in dose volume histograms)^15^. However, Nelms et al.^2^, M. Stasid et al.^16^, and Anna Fredh et al.^17^ found that the dose deviation during clinical practice cannot be properly predicted by the GPs of whole body. According to Heming Zhen et al.^18^, though the distilled GPs of whole body was able to be used to quantitatively evaluate the quantity of dose deviations within the ROI, it cannot provide the intensity information of dose deviation and corresponding spatial information. The acceptance criteria of current patient‐specific QA should be decided based on whether the DVH difference of the current plan meets the clinical treatment objective. M. Stasid et al.^16^, G. Heilemann et al.^19^, and Jinling Yi et al.^20^ found that the GPs of individual volume were more sensitive to dose deviation, so they adopted the GPs of individual volume instead of that of whole body. That can solve the problem of weak correlation between the GPs evaluation result and the clinically relevant metrics to some extent, while removing the limitations to intensity information and spatial information of dose deviation in whole body gamma analysis. Subsequently, M. Cozzolino et al.^21^ proved again that GPs had weak correlation with clinical dose deviation. At the same time, he also recommended that more reference should be made to DVH information in the patient‐specific QA in addition to focusing on the GPs of specific structures. Ruurd Visser et al.^22^ and A. Sdrolia et al. ^23^ completed the dose verification by combining the DVH information and GPs in studies. Their results of studies indicated that the introduction of DVH information indeed improved the correlation between the results of patient‐specific QA and clinically relevant metrics. Therefore, completing dose verification by combining the GPs of individual volume with corresponding DVH information is a better approach to improve correlation between gamma analysis result and clinical criteria.

In fact, Nelms et al.^2^ had proposed the initial concept of achieving the division of evaluation results based on GPs action levels and DVH action levels in his study as early as in 2011. His basis was the “false negative” and “false positive” in 2D verification results of IMRT plans. Also, Ruurd Visser et al.^22^ completed the 3D dose verification by the GI evaluation incorporating DVH information. That achieved the division in responsibility of the medical physicist and radiation oncologist within the QA procedure. From these studies, the introduction of the corresponding DVH information on the basis of the individual volume gamma analysis undoubtedly improved the correlation between the gamma analysis results and clinically relevant metrics. However, Nelms et al.^2^ only proposed the division between “false negative” and “false positive” in 2D verification results in their study. They did not clarify the action levels of DVH information and GPs. In the study of Rusd Visserd et al.^22^, GPs were separated from DVH information, and the action levels of the DVH information were too strict. Therefore, this study aims to accomplish 3D dose verification by incorporating the GPs and DVH information. Then, it proposes appropriate action levels to analyze the non‐negative results in verification of IMRT plan, so that the patient‐specific QA can be completed by properly combining the clinical relevant metrics.

## 2 MATERIALS AND METHODS

### 2.A QA plans

In this study, IMRT treatment plans for 51 patients were selected for dose verification. First, the dose of the 51 IMRT plans was calculated on a 2.5‐mm isotropic dose grid with anisotropic analytical algorithm (AAA) through Eclipse v. 13.5 (Varian Medical Systems, Palo Alto, CA, USA). After that, these plans were delivered by a 6‐MV linear accelerator (Unique, Varian Medical Systems, Palo Alto, CA, USA). The accelerator was equipped with the millennium 120 multileaf collimators. In the dose verification, the structures in all plans were categorized into target volume and organs‐at‐risk (OAR).The details were as shown in Table I.

**Table I:** Details of 51 IMRT Plans

Site	Plans	Fields	Structure – DVH index
Oropharynx	17	9	(GTVnx, GTVnd, PTV) – (D_95_ ^a^, D_mean_ ^b^)
			(Spinal cord, Brainstem) – (D_2cc_ ^c^)
			Parotids – D_mean_
Esophagus	17	5	(PTV, GTV) – D_95_, D_mean_)
			Spinal cord – D_2cc_
			(Heart, Lung) – D_mean_
Pelvic	17	7	PTV – (D_95_, D_mean_)
			(Bladder, Rectum, Femoral heads) – D_mean_

^a^ D_95_ is the dose received by 95% of the volume.

^b^ D_mean_ is the mean dose.

^c^ D_2cc_ is the dose received by 2 cc.

### 2.B QA procedure

Delta4 system (ScandiDos AB, Uppsala, Sweden) was selected as the QA device for dose verification. It was composed of an orthogonal detector array (including a cylindrical PMMA phantom, a main unit and wing units inside the phantom), an inclinometer, ScandiDos Delta4 software, etc. An angle inclinometer was mounted on gantry for the angle response measurement. The ScandiDos Delta4 software measured the dose according to the signals received by the detectors and compared it with the predicted dose. In addition, before the dose verification, the array response calibration and absolute dose calibration of the Delta4 detector array were strictly carried out according to operation manual.

**Fig. 1:**
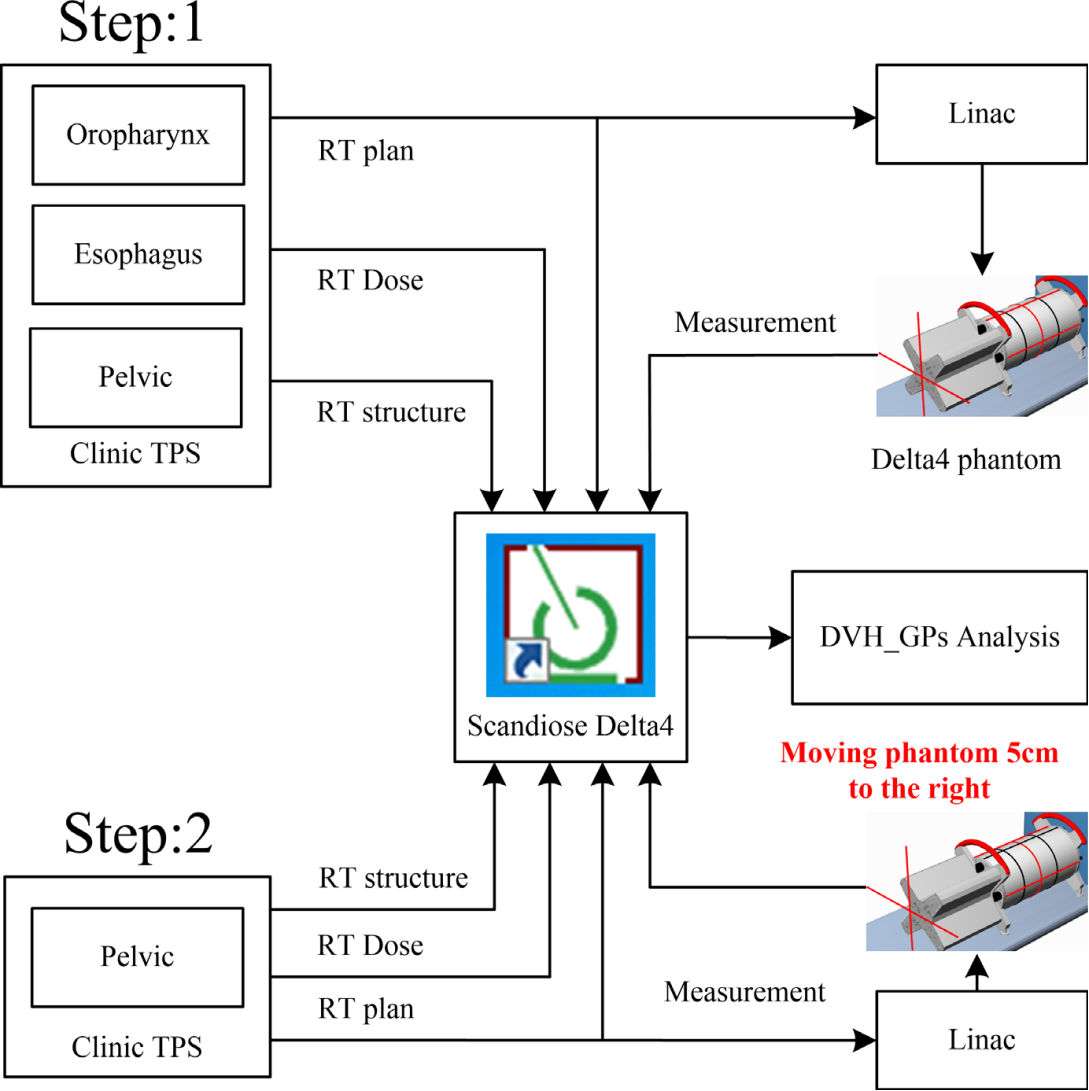
Flowchart of QA procedure

As shown in Figure 1, the QA procedure contained two parts. In the first part, all plans’ isocenter was matched with Delta4 phantom's center. The RT plan and RT dose of these plans were calculated by TPS based on Delta4 phantom. Then the measured dose of these plans on the linear accelerator was acquired by Delta4 system. Finally, these plans were analyzed and compared through the ScandiDos Delta4 software in combination with RT dose, RT plan, and RT structure imported by TPS system. In that way, the DVH information and GPs of specific structures were obtained. As for the second part, the process was basically same except for two distinctions. First, dose verification was only performed to 17 cervical neoplasm plans. Second, when calculating the Delta4 phantom dose, the Delta4 was moved 5 cm to the right by facing Gantry based on the isocenter of the first part, so that the entire left femoral head was completely situated inside of the Delta4 phantom. The shifting of Delta4 phantom was the most important distinction in the two parts.

### 2.C Data analysis

With the help of ScandiDos Delta4, according to the imported RT structure, we obtained specific structures’ GPs and corresponding DVH information based on the criteria of 3%/2 mm (global normalization). Of course, they were the data within the prescription dose coverage region ranging from 10% to 500%. According to Table I, the dose errors (%*DE*) between the predicted dose and measured dose of specific structures’ DVH index were calculated as follows:

$$\%DE=\left|\frac{D_{\mathrm{TPS}}-D_{Delta4}}{D_{Delta4}}\right|\times 100.$$

where, %*DE* is the relative error between the planned dose and the measured dose of DVH index, *D_Delta_*_4_ is the measured dose of the structure under evaluation, and *D_TPS_* is the dose that should be planned.

**Fig. 2:**
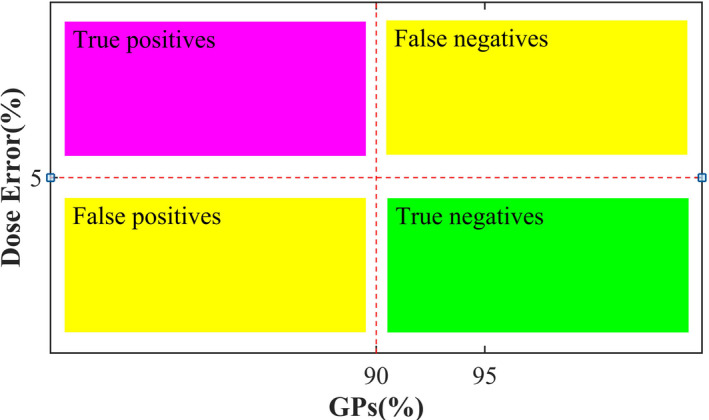
DVH_GPs evaluation chart

Based on the study of the literature^2,22^, the DVH information and gamma passing rates (DVH_GPs) 2D evaluation chart as shown in Figure 2 was built. In this chart, the horizontal axis was the GPs of the current structure under evaluation, and the vertical axis was the %*DE* of DVH index. AAPM TG‐218^14^ report explicitly explained the action limits and tolerance limits of patient‐specific QA. The results out of action limits could result in harm to the patient. The results within the range of tolerance limits indicated that the plan was operated normally. As a consequence, the determination of action levels for DVH index and GPs was the key point of DVH_GPs evaluation method.

First of all, there had been quite a few studies about the action levels of DVH index. M. Cozzolino et al.^21^ and Ruurd Visserc, et al.^22^ set the DVH action levels of 2%–5%, but they concluded that the action levels were too strict for some specific structures such as the target volume and OAR near the target volume. Yi et al.^20^ and Jin et al.^24^ attempted to adopt the DVH‐based action levels of 3% and 5%, respectively, in the 3D gamma analysis. The ESTRO report^25^ recommended an action limit of 5% for IC verification in the IMRT QA. Combined with the above study results, and considering the action limits in dose measurement recommended by AAPM TG‐119 report and TG‐218 report, the DVH action levels in this study was finally set to be 5%. Second, the universal action limits of GPs in gamma analysis were recommended to set to 90% (3%/2 mm and global normalization) in AAPM TG‐218 report^14^. Therefore, the action levels of GPs in this study were also set to 90%.

As described in Figure 2, the action levels of the vertical axis %DE were 5%, while the action levels of the horizontal axis GPs were 90%. In addition, the tolerance levels of GPs were set to 95% in order to better investigate the QA results. The entire DVH_GPs evaluation results were divided into four regions, including the true positive (TP) (%*DE*＞5%, GPs＜90%), the false positive (FP) (%*DE* ≤ 5%, GPs＜90%), the false negative (FN) (%*DE*＞5%, GPs ≥ 90%), and the true negative (TN) (%*DE* ≤ 5%, GPs ≥ 90%). Combined with the DVH_GPs evaluation results, whether the current plans were acceptable for treatment must be considered from a clinical point of view. That was to say, under comprehensive analysis of all factors, the top priority for pass/fail decisions should be given to DVH difference^18^.

## 3 RESULTS

As shown in Tables II, the average GPs with 3%/2 mm criterion of whole body was more than 95%, meeting the universal tolerance limits of TG‐218 report. However, in the organ structure oriented DVH_GPs evaluation, 25 cases with FN, 5 cases with TP, and 2 cases with FP were observed in oropharynx neoplasm plans. 20 cases with FN were observed in esophageal neoplasm. 33 cases with FN were observed in cervical neoplasm plans. In these results, extreme abnormal FN results were observed in structures as follows: (i) lung (%*DE* = 21.52%±8.20%) and heart (%*DE* = 19.76%) of oropharynx neoplasm plans; and (ii) bilateral femoral heads (%*DE* = 26.41%±13.45%) of cervical neoplasm plans. After performing Delta4 phantom shift verification, extreme abnormal FN results were hardly observed. Finally, according to clinical practice, only one case with TP in oropharynx neoplasm plans and one case with FN in esophageal neoplasm plans did not meet the treatment requirements, and needed to be replanned.

**Table II:** Statistical results of DVH_GPs evaluation for all the plans

Site	GPs of whole body (Mean + STD)	QA decision:Total (Fail)			
		TN	FN	FP	TP
Oropharynx	98.01%±2.46%	87 (0)	25 (0)	5 (0)	2 (2)
Esophagus	99.60%±0.62%	64 (0)	22 (1)	0 (0)	0 (0)
Pelvic	99.63%±0.95%	52 (0)	33 (0)	0 (0)	0 (0)
Total	99.08%±1.71%	203 (0)	58 (1)	5 (0)	2 (1)

DVH_GPs, DVH information and gamma passing rates; TN, true negative; FN, false negative; FP, false positive; TP, true positive.

### 3.A QA results when isocenter situated in the center of Delta4 phantom

#### 3.A.1 Evaluation results of oropharynx neoplasm plans

**Fig. 3:**
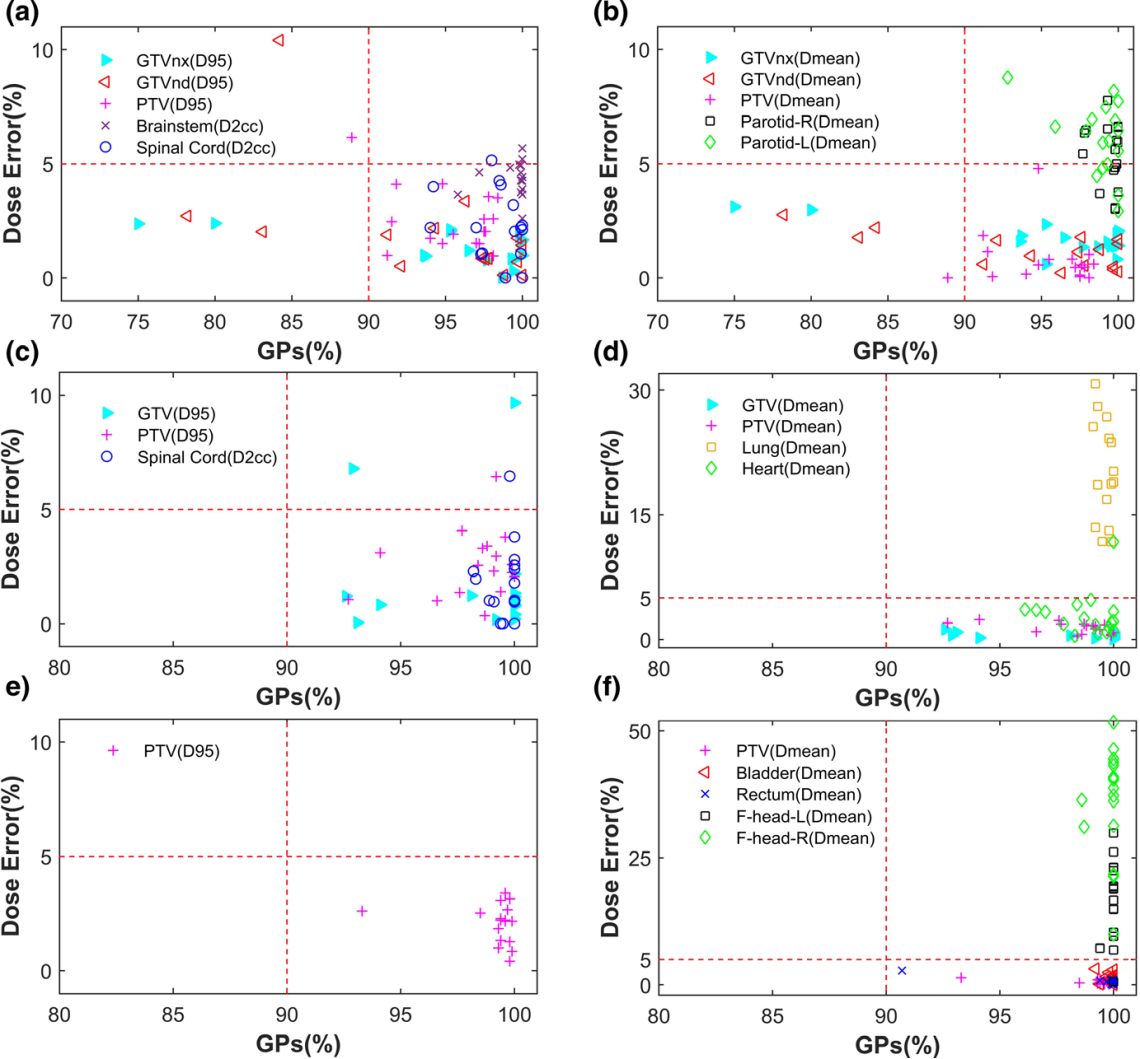
DVH_GPs evaluation results in all the plans

Figure 3(a) and 3(b) showed the DVH_GPs evaluation results of the 17 oropharynx neoplasm plans. As for the target volume, 1 case with TP was observed in PTV; 2 cases with FP were observed in GTVnx; 3 cases with FP and 1 case with TP were observed in GTVnd. As for the OAR, 1 case with FN was observed in the spinal cord, 2 cases with FN were observed in the brain stem; 22 cases with FN were observed in bilateral parotid glands.

In QA decision, first, the %*DE*_D95_ and GPs of GTVnd TP case were 10.41% and 84.2%, respectively, and they both did not meet the patient‐specific QA requirements. Therefore, this plan needed to be replanned. Second, as for the 2 FP cases in GTVnx and 3 FP cases in GTVnd, their GPs were significantly lower than the normal level (only 80%, 75%, 83.1%, 78.2%, 61.9%, respectively). But their %*DE* were within the range of action limits (%*DE*_D95_ = 2.88%±1.03%, %*DE*_Dmean_ = 2.37%±0.74%). So, they were accepted for treatment. Third, there were 1 TP case in PTV (%*DE*_D95_=−6.15%, GPs = 88.9%), 1 FN case in spinal cord, and 2 FN cases in brain stem. Their %*DE* were all higher than 5%. But after analyzing the signs of these dose differences, they were acceptable from the clinical and radiological point of view. Finally, the %*DE*_Dmean_ of the parotid glands were higher than 5% (%*DE*_Dmean_ = 5.74%±1.51%). However, the parotid glands were situated inside the region with high‐dose gradient, so all the DVH_GPs evaluation results of parotid glands were also acceptable in clinical treatment.

#### 3.A.2 Evaluation results of esophageal neoplasm plans

Combining the statistical results in Table II with the DVH_GPs evaluation chart in Figure 3(c) and 3(d), as for target volume, one case with FN result was observed in PTV, two cases with FN result were observed in GTV. As for OAR, one case with FN result was observed, respectively, in spinal cord and heart, and FN results were observed in lung of all plans.

First, in the FN results of PTV and GTV, the %*DE*_D95_ were −6.43%, −9.67%, and 6.79%, respectively. In addition, the %*DE*_D2cc_ in the FN result of spinal cord was 6.45%. These results were clinically acceptable and the corresponding plans passed the dose verification except for the FN case of GTV whose %*DE*_D95_ was 6.79%. Because the %*DE* value of 6.79% indicated that the measured does was less than the predicted dose. And that illustrated that the corresponding plans did not meet the tumor's local control requirement and needed to be replanned. Moreover, the heart's FN result was just the opposite, with a %*DE*_Dmean_ as high as −11.76%. Its %DE severely exceeded the DVH dose tolerance requirement. Therefore, the heart evaluation result was unacceptable in clinical treatment and a further analysis was required. More importantly, in the lung evaluation results of all plans, their %*DE*_Dmean_ were all higher than 10% and as high as 44.22%. Therefore, the DVH_GPs evaluation results of lung in all plans were unacceptable for clinical treatment and they also needed further evaluation and analysis.

#### 3.A.3 Evaluation results of cervical neoplasm plans

According to DVH_GPs of cervical neoplasm as shown in Figure 3(e) and 3(f), TN results were observed in PTV, rectum, and bladder. There were 33 cases with FN results in femoral heads.

The %*DE*_Dmean_ of left and right femoral heads were as high as 16.60%±7.69%, 36.21%±10.48%, respectively, and that severely exceeded the required DVH dose tolerance. Therefore, the evaluation results of femoral heads of these plans were unacceptable for clinical treatment, and they also required further analysis.

### 3.B QA results after shifting of Delta4 phantom

In DVH_GPs evaluation, the %*DE*_Dmean_ of the lung and heart in the esophageal neoplasm plans and of the femoral heads in cervical neoplasm plans were severely deviated from the normal value. The relevant parameters of these specific structures shown in Table III were collected, and the cause of abnormal %*DE*_Dmean_ was further analyzed. Besides providing the DVH information and GPs of these structures, ScandiDos Delta4 software also provided their volumetric parameters *V*_tot_ and *V*_used_, etc.

**Table III:** Statistical data of structures with significant %*DE* in DVH_GPs evaluation

Structure	Number	*V*_used_ ^a^/*V*_tot_ ^b^	GPs	%DE
Heart	1	0.08	100	19.76
Lung	17	0.55 ± 0.23	99.83 ± 0.20	21.52 ± 8.20
Femoral head	34	0.42 ± 0.25	99.98 ± 0.10	26.41 ± 13.45

^a^ *V*_used_ indicates the part of *V*_tot_ that lies inside the calculation volume.

^b^ *V*_tot_ indicates the total volume of the selected structure (including the parts outside the calculation volume and outside the phantom).

**Fig. 4:**
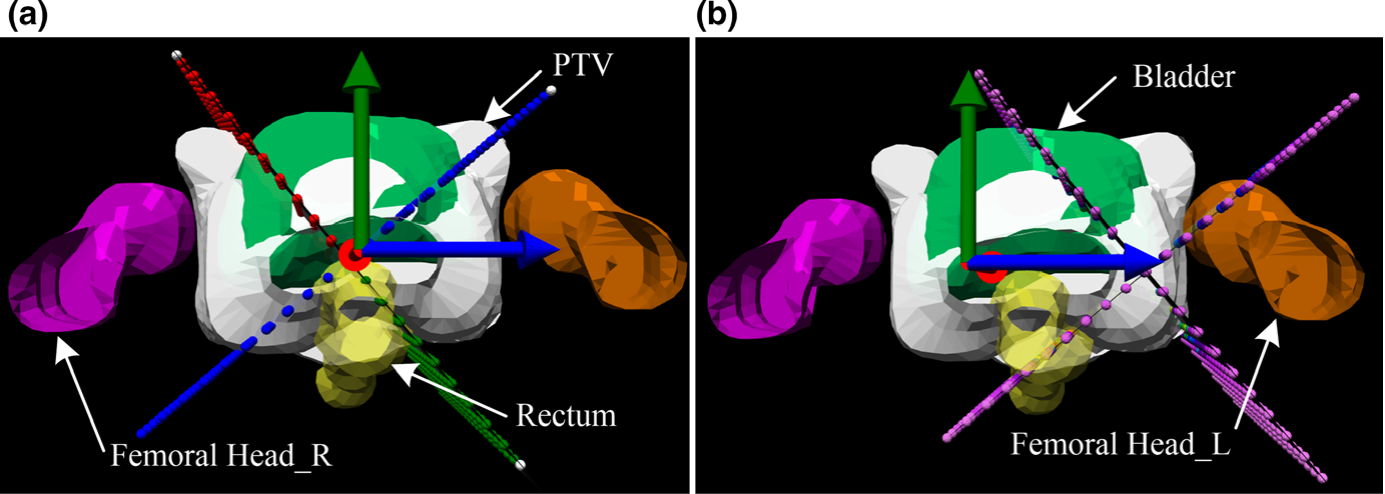
Shift of Delta4 phantom

Through observation, there was inconsistency between *V*_used_ and *V*_tot_ of these structures. This may be the cause for the significant FN results of these structures. To further confirm the assumption, taking the left femoral head with obvious *V*_used_/*V*_tot_ difference (0.22 ± 0.12) as an example, Delta4 phantom was shifted 5cm horizontally to the right by facing gantry, so that the left femoral head was completely covered inside the phantom (as shown in Figure 4). Then, these plans were reverified and the DVH_GPs evaluation was re‐executed.

To confirm whether *V*_used_/*V*_tot_ had an impact on %*DE*_Dmean_ and GPs, *V*_used_/*V*_tot_, %*DE* and GPs before and after the shifting were collected. Then, the two‐tailed paired‐sample T tests were carried out to the two sets of data, and a significant difference was found when *p < *0.05. According to the results as shown in Table IV, the revised *V*_used_/*V*_tot_ of the left femoral head was significantly different from original data. And *V*_used_ was basically equal to *V*_tot_ after the shifting. Meantime, the revised %*DE*_Dmean_ of the left femoral head was also different from the original data. After the shifting, the significant FN results in the left femoral head nearly disappeared and the average %*DE*_Dmean_ was less than 3%. In addition, no significant difference was found in GPs of the left femoral head between original data and revised data.

**Table IV:** Results of two‐tailed paired‐sample T tests of the left femoral head before and after phantom shift

Index	Origin	Revised	Mean difference	*P*
	(Mean ± STD)			
*V*_used_/*V*_tot_	0.22 ± 0.12	0.93 ± 0.10	‐0.71 ± 0.13	0.00
%*DE*_Dmean_	16.60%±7.69%	2.98%±1.57%	13.62%±7.58%	0.00
GPs	99.96%±0.15%	99.99%±0.05%	‐0.02%±0.16%	0.54

**Fig. 5:**
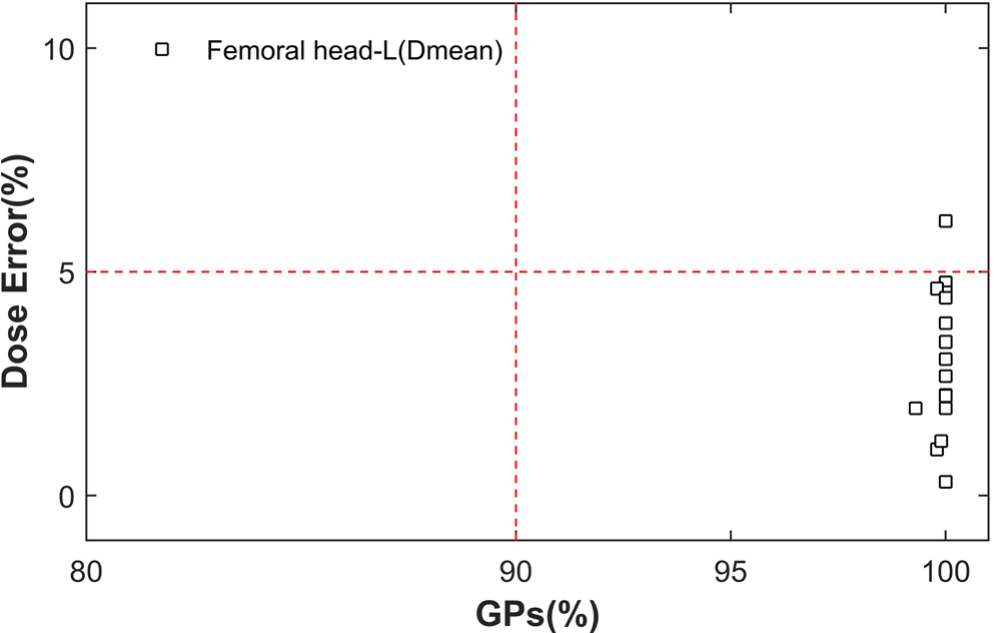
DVH_GPs evaluation results of femoral head after the shift of Delta4 phantom

Then, DVH_GPs evaluation was carried on to the left femoral head after the shifting of the phantom. As shown in Figure 5, only one case was still with FN in the left femoral head in the evaluation results. The %*DE*_Dmean_ of the left femoral head of this plan was −6.13%. As for D_mean_ in the femoral head, though the actually measured dose was higher than the predicted dose, it was far lower than the limit value. Therefore, this case was acceptable for the clinical treatment. In general, after the shifting, the significant FN of the left femoral head nearly disappeared, and the evaluation results of the left femoral head were all acceptable for clinical treatment.

Based on above results, the similar phantom shifting dose verifications were carried out to the remaining structures with significant different %*DE*. All FN results with severe %*DE* difference returned to normal levels. It was acceptable for the DVH_GPs evaluation results of all these structures from clinical point of view.

## 4 DISCUSSION

Gamma analysis can condense the verification measurements into a single value (GPs). Reviewing QA result by GPs has both advantage and disadvantage. The advantage is that GPs improves the efficiency of dose verification under the current busy clinic. The disadvantage is that most of patient‐specific QA is only limited to the percentage of failed points. However, the distilled GPs values cannot provide dose intensity information and the corresponding location information of failed points^26^, ^27^. That is why gamma analysis’ capability in detecting clinically significant deviations has been questioned widely. As presented in Figure 3 and Table II, the average GPs of whole body (3%/2 mm) for three types of IMRT plans were 98.01%±2.46%, 99.60%±0.62%, 99.63%±0.95%, respectively. However, large dose difference was observed in some plans. And in these plans, their GPs were all higher than the universal action limits of 90%, especially the universal tolerance limits of 95% recommended in AAPM report TG‐218. These results also proved GPs’ weak correlation to clinical dosimetric difference in IMRT QA^2^, ^18^, ^24^. Therefore, a simple gamma analysis is no longer applicable, and patient‐specific QA decisions must be linked with DVH information. With the DVH_GPs evaluation method, the individual volume gamma analysis was able to provide the approximate location information of the specific structure under evaluation. The DVH information of the corresponding structure can provide the dose intensity information related to clinical treatment criteria. More importantly, the introduction of DVH information can improve the correlation between the patient‐specific QA results and clinically relevant metrics. Of course, the focus of DVH_GPs evaluation method was how to make the final QA decision in combination with clinical treatment criteria.

In our study, when DVH action levels were set to 5% and GPs action levels were set to 90%, a considerable quantity of non‐negative results were observed in DVH_GPs evaluation. However, only two cases did not meet the clinical treatment requirements, and their plans needed to be replanned. First, as an important tool for evaluating the plan's feasibility for clinical treatment, DVH index shall be the primarily concerned parameter for the patient‐specific QA determination^27^. It is emphasized in AAPM TG‐218 report that DVH analysis can be used to evaluate the clinical relevance of the QA results, especially when the GPs fails the tolerance limits or is inconsistent. In DVH analysis, apart from considering the strength of %*DE*, it was also essential to analyze the sign. For results whose %*DE* exceeded DVH action levels, it was acceptable from clinical point of view if the target volume's actual dose is higher than the predicted dose or if the measured dose of OAR was lower than the predicted dose. Second, although GPs had a very weak correlation with clinical treatment criteria, it was still an important index for evaluating TPS system and delivery system. It is recommended in AAPM TG‐218 report^14^ that gamma statistics should be provided in a structure by structure basis and gamma distribution should be carefully reviewed rather than only relying on distilled statistical evaluations. The 5 FP cases of oropharynx neoplasm plans were derived from target volume. The lower GPs of target volume were partly ascribed to the fact that failed points were mainly clustered in high‐dose region^28^, ^29^. Another reason was the reduction of evaluation volume. FP case can indicate that a IMRT plan does not have adverse effect, but that does not mean the lower GPs should be ignored. For this reason, on the premise of ensuring %*DE* within the DVH action limits, GPs should be also carefully investigated. In addition, the features of specific structure also generated some non‐negative results, such as the parotid gland in the region with high‐dose gradient, etc. The cause was that the dose model of the penumbra region within the gradient region did not have the required accuracy^22^. Therefore, for this type of specific structures, it was acceptable if %*DE* of D_mean_ was slightly more than 5%. For low‐dose regions (e.g., lung, femoral head), as the gamma analysis adopted the global normalization, it was easy to mask the significant dose difference in this region^18^, ^30^. Of course, the large quantity of FN cases in lung and femoral heads in the study may also be caused by other two reasons besides the dose normalization. One reason was the significant difference between *V*_used_ and *V*_tot_ due to the limitation of size of the Delta4 phantom. The other reason was the over‐travelled fields with less accurate dose calculation implemented by the TPS^22^ because the specific structures were far away from the isocenter. In addition, the complex bifurcating target volumes separated by large area of low‐dose region may also result in lower GPs or high %DE^10^ (such as TP case in the oropharynx neoplasm plans). In general, in addition to considering the DVH index and GPs, the final evaluation results were closely linked with the features of the specific structure, the treatment site, the QA equipment, the delivery technique and so on^31^, ^32^, ^33^, ^34^. Therefore, it is necessary for the medical physicist to carry out an objective analysis based on the actual situation, and to correctly deal with the non‐negative results in DVH_GPs by combining clinical treatment criteria.

Of course, the DVH_GPs evaluation method proposed in the study was limited by QA equipment, delivery system, tumor sites, complexity of IMRT plans, action levels’ setting, and so on. The 5% DVH action levels and 90% GPs action levels may be not suitable for all the structures. Therefore, an interesting alternative for future research could be calculating action limits separately for different complexity plans and different structures. Although DVH_GPs evaluation method could cause patient‐specific QA less efficient, it can increase insight into dose delivery to patient‐specific structures and can be used to make an objective decision based on clinically relevant dose differences.

## 5 CONCLUSIONS

By adopting the individual volume gamma analysis, the DVH_GPs evaluation method is able to make up the disadvantage of lacking the dose intensity information and position information of the conventional simplex GPs evaluation. The DVH information is directly related to clinical treatment; hence it enhances the correlation between the results of Patient QA and the clinically relevant metrics. In our study, by setting 5% DVH action levels and 90% GPs action levels for DVH_GPs analysis, we were able to further reveal the TP, FR, TN, and FN indicators in the evaluation results. The evaluation results were affected by many factors. Therefore, the medical physicist should keep more focus on DVH information and not be limited in the distilled GPs in patient‐specific QA. Besides, current actual clinical conditions should be taken a full consideration in the final QA acceptance determination.

## CONFLICT OF INTERESTS

The authors declare no conflict of interest.
